# Hepatoprotective Effect of Meroterpenoid-Rich Fraction from the Ethanolic Extract of *Sargassum sarratifolium* on *tert*-Butyl Hydroperoxide-Induced Mice

**DOI:** 10.4014/jmb.2501.01014

**Published:** 2025-05-27

**Authors:** Sujin Lim, Ji Young Hwang, Kyoung Mi Moon, Hyeon Hak Jeong, Jaeseong Seo, Hyeung-Rak Kim, Bonggi Lee

**Affiliations:** 1 Department of Food Science and Nutrition, Pukyong National University, Busan 48513, Republic of Korea; 2 Department of Smart Green Technology Engineering, Pukyong National University, Busan 48513, Republic of Korea; 3 Marine Integrated Biomedical Technology Center, The National Key Research Institutes, Pukyong National University, Busan 48513, Republic of Korea

**Keywords:** Oxidative stress, hepatoprotection, *Sargassum serratifolium*

## Abstract

The liver, critical for detoxification and metabolic homeostasis, is highly susceptible to oxidative stress caused by reactive oxygen species (ROS). This study evaluated the hepatoprotective effects of a meroterpenoid-rich fraction (MES) from *Sargassum serratifolium* in a tert-butyl hydroperoxide (t-BHP)-induced oxidative liver injury model in mice. MES treatment ameliorated t-BHP-induced histopathological changes, including hepatocyte swelling, cytoplasmic vacuolation, and necrosis. Biochemical analyses showed that MES significantly decreased serum alanine aminotransferase (ALT) and aspartate aminotransferase (AST) activities to 70% and 50%, respectively, of the levels observed in the t-BHP-treated group. It also reduced Lactate Dehydrogenase (LDH) activity, which was increased fivefold by t-BHP, by up to 70%, and significantly reduced hepatic Malondialdehyde (MDA) levels. MES restored the activities of antioxidant enzymes including Superoxide Dismutase (SOD), catalase, Glutathione Peroxidase (GPx), and Glutathione Reductase (GR), and activated the Nuclear factor (erythroid-derived 2)-like 2 (Nrf2) pathway, leading to increased glutathione (GSH) levels and enhancing the expression of detoxifying enzymes such as heme oxygenase-1 (HO-1) and NAD(P)H:quinone oxidoreductase 1 (NQO1). These findings demonstrate that MES provides effective protection against oxidative liver damage by mitigating oxidative stress, enhancing antioxidant defenses, and activating the Nrf2 signaling pathway. This highlights the potential of marine-derived natural products as hepatoprotective agents.

## Introduction

The liver is a vital organ primarily responsible for detoxifying xenobiotics and maintaining metabolic homeostasis, making it highly susceptible to damage from various sources such as environmental pollutants, chemicals, alcohol, drugs, and pathogenic infections [1]. Such harmful agents often lead to the overproduction of reactive oxygen species (ROS) during metabolic processes, subsequently causing oxidative stress, cellular dysfunction, apoptosis, necrosis, and ultimately, liver disease [2-4].

ROS, a natural byproduct of aerobic respiration, plays a dual role within biological systems. While essential for host defense mechanisms against invading pathogens [5], ROS can also activate cellular signaling pathways that govern cell growth and death [2]. However, when produced excessively, ROS contribute to oxidative stress, damaging critical biomolecules like DNA, proteins, and lipids, which can lead to degenerative diseases including cardiovascular disorders, atherosclerosis, and cancer [6, 7]. Oxidative stress is also a key driver of liver injury.

Cellular defense against oxidative stress involves enzymatic antioxidants such as superoxide dismutase (SOD), catalase, and glutathione peroxidase (GPx), and non-enzymatic compounds including glutathione (GSH), vitamin C, and vitamin E [8]. Among regulatory pathways, nuclear factor (erythroid-derived 2)-like 2 (Nrf2) plays a central role in activating genes that encode detoxifying and antioxidant enzymes in response to oxidative stimuli [9, 10]. This pathway is particularly relevant in the context of natural product-based hepatoprotection.

Tert-butyl hydroperoxide (t-BHP) is widely used in research to model liver injury induced by oxidative stress, as it is metabolized in hepatocytes through pathways involving cytochrome P450 and GPx, leading to the production of reactive intermediates that cause lipid peroxidation and cellular damage [11, 12].

Sargassum, a genus of brown algae known for its diverse bioactive compounds including meroterpenoids, phlorotannins, and fucoxanthin, has been used in traditional medicine and has demonstrated significant anti-inflammatory, anti-cancer, and hepatoprotective properties [13-15]. Among brown algae, *Sargassum serratifolium* is particularly rich in structurally unique meroterpenoids, including sargahydroquinoic acid (SHQA), sargachromanol (SCM), and sargaquinoic acid (SQA). These compounds have demonstrated notable anti-inflammatory activity compared to meroterpenoids found in other species, making *S. serratifolium* a promising candidate for inflammation-related liver protection. [16].

Given the promising bioactive profile of the meroterpenoid-rich fraction (MES) isolated from the ethanolic extract of Sargassum serratifolium, this study aims to explore its hepatoprotective effect against t-BHP-induced liver injury in mice, potentially offering a natural therapeutic strategy against oxidative liver damage.

## Materials and Methods

### Preparation of MES and Isolation of Chemical Compounds

Sargassum serratifolium specimens were collected from the coast of Busan, South Korea, in May 2017. The identity of these specimens was authenticated by algal taxonomist C.G. Choi from the Department of Ecological Engineering at Pukyong National University, Republic of Korea. Subsequently, the gathered samples underwent air-drying and grinding processes to facilitate extraction. A total of 1.5 kilograms of dried sample underwent sequential extraction with 70% ethanol (6 liters each time) at 70°C for 3 h. The resultant combined extract underwent filtration using an ultrafiltration unit with a molecular weight cutoff of 50 kDa, followed by concentration until the lipophilic fraction was isolated from the salt water. The lipophilic fraction was dissolved in ethanol, mixed with an equal volume of distilled water, and concentrated using a rotary vacuum evaporator (Eyela N3010, Japan). This process of dissolution and washing was repeated five times. From the initial 1.5 kilograms of dried sample, 120 grams of the meroterpenoid-rich fraction (MES) was obtained and utilized for subsequent experiments.

### Animal Treatment

Male ICR mice, aged 6 weeks with a body weight of 29.5 ± 2.5 g, were acquired from Central Laboratory Animal Inc. (Republic of Korea). The mice were housed with ad libitum access to food and water and maintained in a controlled environment at 21 ± 2°C and 50 ± 5% relative humidity, under a 12-h light/dark cycle. The mice were acclimatized for one week before the commencement of the experiments. Thirty-six healthy male mice were randomly assigned to six groups (6 mice per group). To investigate the protective effects of MES against t-BHP-induced acute hepatic damage, 50 mg of MES dissolved in 40% propylene glycol (final concentration 10 mg/ml) was orally administered at dosages of 7 and 14 mg/kg once daily for five consecutive days. Three hours after the final administration, the animals received a single intraperitoneal injection of t-BHP (1.5 mM/kg). Eighteen hours later, for western blotting, the mice were euthanized and liver was excised and immediately frozen in a nitrogen tank. Additionally, for staining, the liver tissues were fixed in 4% paraformaldehyde and embedded in paraffin using Tissue-Tek Optimum Cutting Temperature compounds (Sakura Finetek, USA). All animal experiments were conducted in strict accordance with the guidelines from the Korean National Institute of Health Guide for the Care and Use of Laboratory Animals, and the experimental protocol was approved by the Animal Ethics Committee of Pukyoung National University (Approval No. 2018-08).

### Hepatotoxicity Assessment

Blood samples were centrifuged, and the serum obtained was stored at -20°C for subsequent analyses. Serum levels of hepatic enzymes, alanine ALT and AST, served as biochemical markers for acute hepatic damage. The concentrations of ALT and AST were measured using specific assay kits (Asan Pharm, Republic of Korea). Additionally, lactate dehydrogenase (LDH) activity, an indicator of hepatic cell death, was assessed using an LDH assay kit (Biomax, Republic of Korea) following the manufacturer’s instructions.

### Measurement of Lipid Peroxidation

To measure lipid peroxidation, liver tissues were homogenized in a lysis buffer consisting of 50 mM Tris-HCl (pH 7.5), 150 mM NaCl, 1% NP-40, 1% Tween-20, 0.1% SDS, 1 mM Na_3_VO_4_, 10 μg/ml leupeptin, 50 mM NaF, and 1 mM PMSF. The homogenates were then centrifuged at 10,000 ×*g* for 15 min at 4°C. The resulting supernatant was subjected to lipid peroxidation analysis.

Lipid peroxidation was quantified by measuring the levels of malondialdehyde (MDA), a marker of oxidative stress, utilizing the thiobarbituric acid reactive substances (TBARS) assay. This involves the formation of a MDA-TBA adduct under high temperature and acidic conditions. The MDA concentration was determined using a TBARS assay kit (Cayman, USA), following the manufacturer’s protocol, and measurements were taken with a microplate reader (GloMax-multi detection system, Promega) at 540 nm. MDA levels were normalized to protein content to account for variations in tissue sample sizes.

### Determination of Glutathione Level

Liver tissues were finely chopped and homogenized in 50 mM 2-(N-morpholino) ethanesulfonic acid (MES) buffer supplemented with 1 mM EDTA. The homogenate was then centrifuged at 10,000 ×*g* for 15 min at 4°C. The resulting supernatant was utilized for the GSH concentration using a GSH assay kit (Cayman). Additionally, the activities of antioxidant enzymes, including SOD, catalase, glutathione S-transferase (GST), GPx, and glutathione reductase (GR), were measured using respective activity assay kits (Cayman), in accordance with the manufacturer’s instructions.

### Western Blotting

Western blot analysis was performed based on previously published studies [17]. In brief, 30 μg of proteins were separated by SDS-PAGE and transferred onto a nitrocellulose membrane (Millipore, USA). The membrane was then blocked with 5% skim milk buffer (10 mM Tris, 100 mM NaCl, 0.1% Tween 20) and incubated with primary antibodies. The primary antibodies used were: HO-1 (ab13248) and NQO1 (ab34173) from Abcam (UK); Nrf2 (sc365949), β-actin (sc47778), GST (sc138), and catalase (sc271803) from Santa Cruz Biotechnology (USA). Following incubation with HRP-conjugated secondary antibodies at 25°C for 1 h, the membrane was washed with TBST buffer. Protein detection was conducted using an ECL detection kit, and densitometric analysis was performed using a CCD camera system (EZ-Capture II, ATTO Co., Japan) and CS Analyzer version 3.00 software to quantify protein expression levels. In the “Western blot analysis” section, all scientific content, statistical results, and antibody information were retained. Only redundant phrases were condensed for clarity.

### Histological Analysis

Brifely, sliced liver tissue samples at a thickness of 6 micrometers subsequently stained with hematoxylin and eosin (H&E). Images of the stained sections were captured using a Motic BA210E microscope (Motic China Group Co., Ltd., China) equipped with Motic Images Plus 2.0 software (Motic China Group Co).

### Statistical Analysis

All data were presented as mean ± standard deviation (SD) based on a minimum of three independent experiments, unless specified otherwise. Statistical comparisons were performed using one-way ANOVA followed by Duncan's multiple range test at a 95% confidence level.

## Results

### Histopathology of Liver

**Fig. 1:**
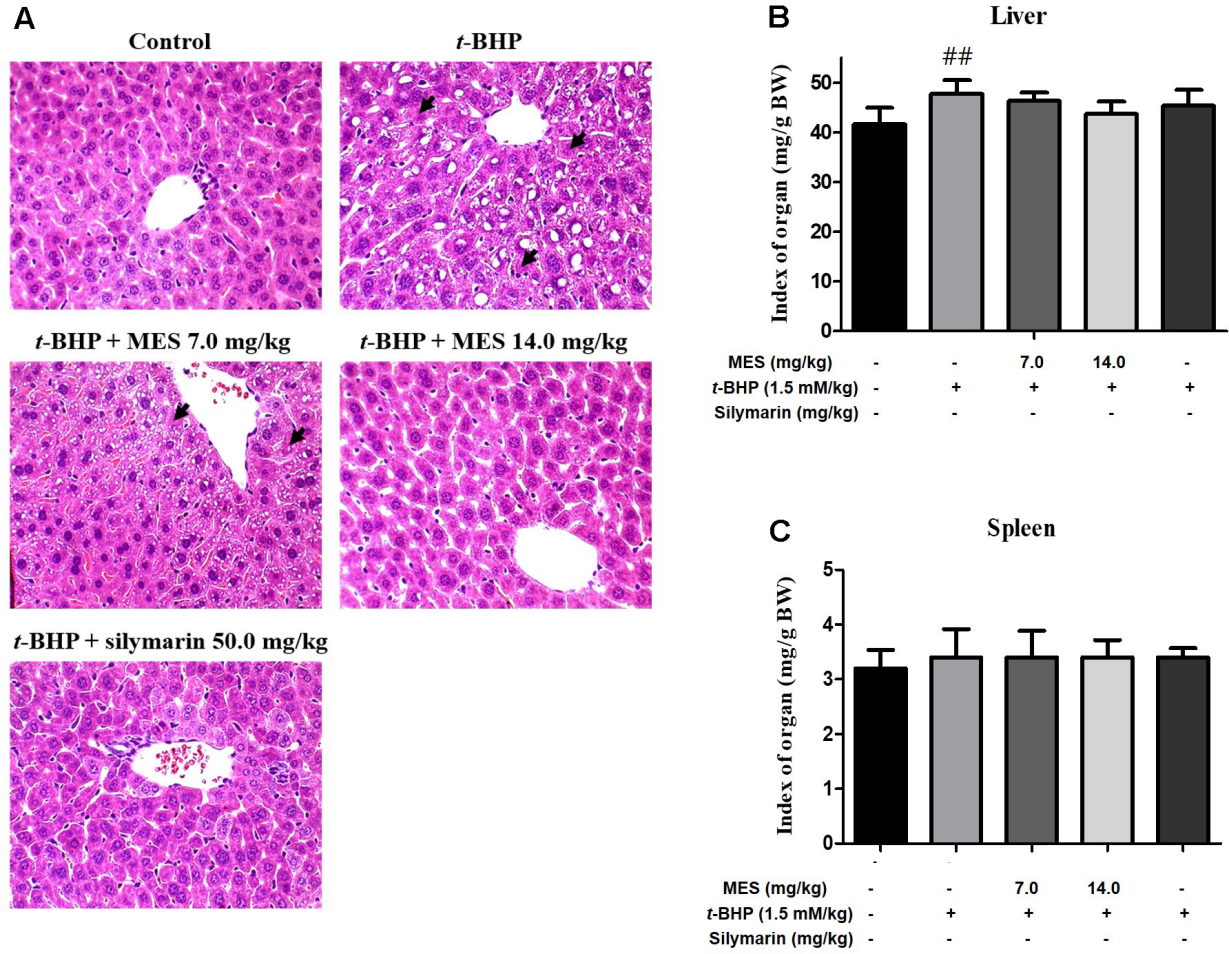
Impact of MES on t-BHP-induced liver damage (**A**) Relative liver weights in t-BHP-treated mice after 5 days of treatment with or without MES or silymarin. (**B**) Liver sections stained with hematoxylin and eosin (×100) from mice (n = 6/ group) pretreated with MES or silymarin for 5 days, then treated with t-BHP (1.5 mM/kg, ip) three hours after the final administration. The t-BHP treatment group showed severe coagulative necrosis (indicated by arrows) and disrupted structural integrity. (**C**) Relative spleen weights in t-BHP-treated mice with or without MES or silymarin treatment for 5 days. Data shown as means ± SD from six mice. ##P < 0.01 indicates significant difference from the control group not treated with t-BHP.

Histopathological analysis was conducted on liver tissues to evaluate the hepatoprotective effects of the MES from *Sargassum serratifolium* against t-BHP-induced hepatotoxicity. Following t-BHP administration, hepatocytes exhibited swelling, vacuolation of the cytoplasm, and necrosis primarily in the portal region of the liver (indicated by arrows in Fig. 1A). Treatment with 7.0 mg/kg of MES or silymarin ameliorated these changes, notably reducing hepatocyte swelling (Fig. 1A). Moreover, MES administration significantly decreased cytoplasmic vacuolation compared to the t-BHP-only treated group, particularly in the portal area (Fig. 1A). Higher doses of MES (14.0 mg/kg) and silymarin showed a more pronounced suppression of hepatotoxicity (Fig. 1A). In terms of organ weights, there were slight but not statistically significant differences in liver weights between the t-BHP-treated group and the control group. Additionally, spleen weights did not show significant differences across all experimental groups (Fig. 1B and 1C).

### Effect of MES on *t*-BHP-Induced Hepatic Damage

**Fig. 2:**
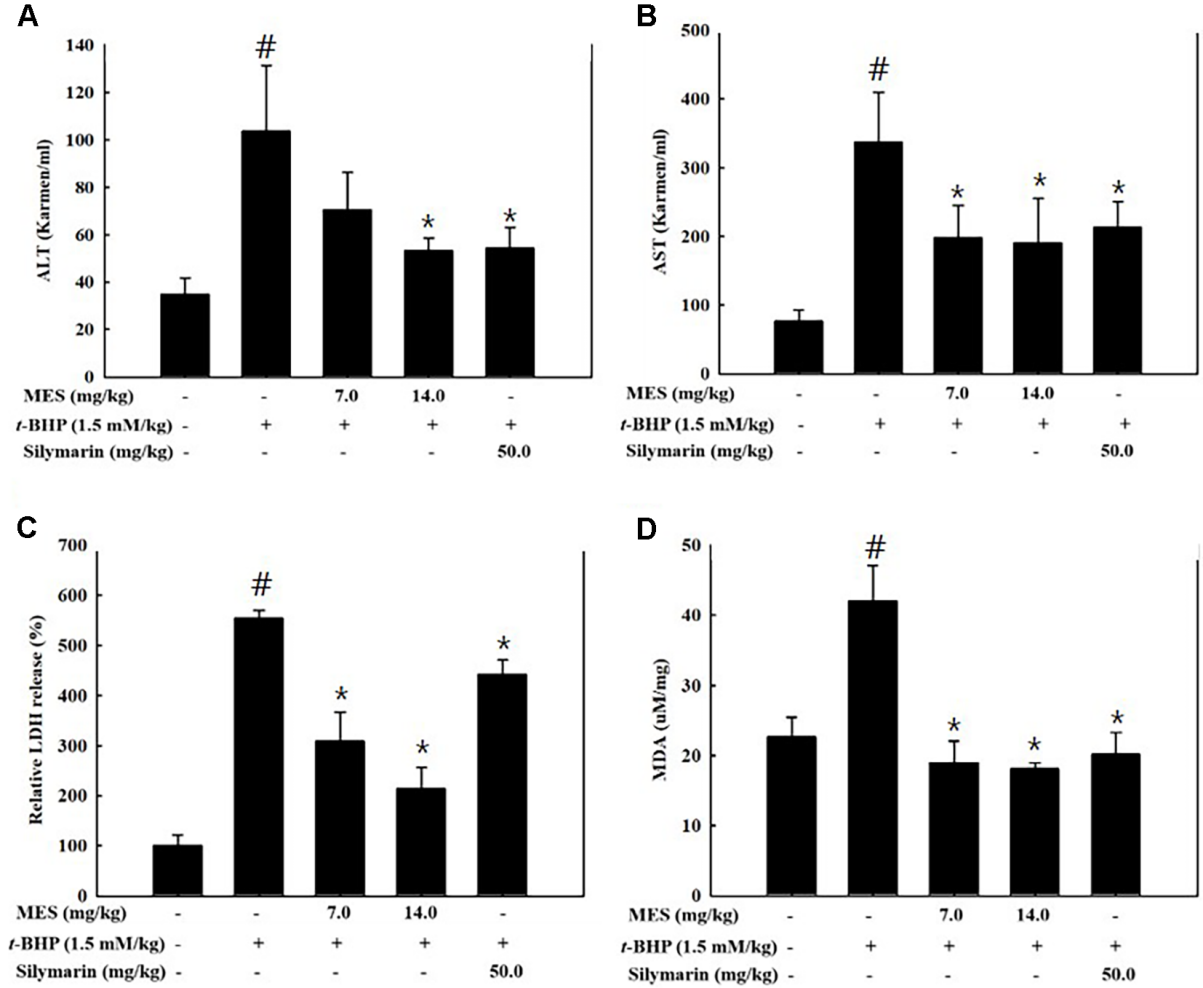
Effect of MES on hepatic enzyme activities and lipid peroxidation in t-BHP-induced mice After 5 days of pretreatment with MES or silymarin, mice (n = 6/group) were administered t-BHP (1.5 mM/kg, ip). Postsacrifice, serum activities of ALT (**A**), AST (**B**), and LDH (**C**) were quantified using respective assay kits. Lipid peroxidation in liver tissues was measured using a TBARS assay kit (**D**). Values are presented as means ± SD. #P < 0.05 indicates significant difference from the control group without t-BHP treatment; *P < 0.05 indicates significant differences from the t-BHP only group.

Serum levels of hepatic enzymes (ALT, AST, and LDH) were evaluated as biochemical markers for acute hepatic damage. Treatment with t-BHP resulted in a threefold increase in both ALT (Fig. 2A) and AST (Fig. 2B) activities compared to the control group. However, treatment with MES or silymarin effectively reduced the activities of these enzymes. Specifically, supplementation with 14.0 mg/kg of MES decreased ALT and AST activities to 70%and 50%, respectively, of the levels observed in the t-BHP-treated group (Fig. 2A and 2B). Additionally, LDH activity was elevated 5-fold following t-BHP treatment, but MES administration dose-dependently mitigated this increase. A high dose of MES (14.0 mg/kg) reduced the t-BHP-induced LDH activity by 70% (Fig. 2C). These findings indicate that MES effectively prevented hepatic damage induced by t-BHP-associated oxidative stress. Silymarin, used as a positive control, also diminished the t-BHP-induced increases in AST and ALT activities, though its reduction of LDH activity was less pronounced (Fig. 2A-2C). Moreover, t-BHP treatment led to enhanced lipid peroxidation in the liver, as evidenced by doubled MDA levels. Conversely, both MES and silymarin treatments significantly lowered MDA levels compared to those in the control group (Fig. 2D), further corroborating their protective effects against oxidative hepatic injury.

### Effect of MES on Glutathione Level in *t*-BHP-Induced Mice

**Fig. 3:**
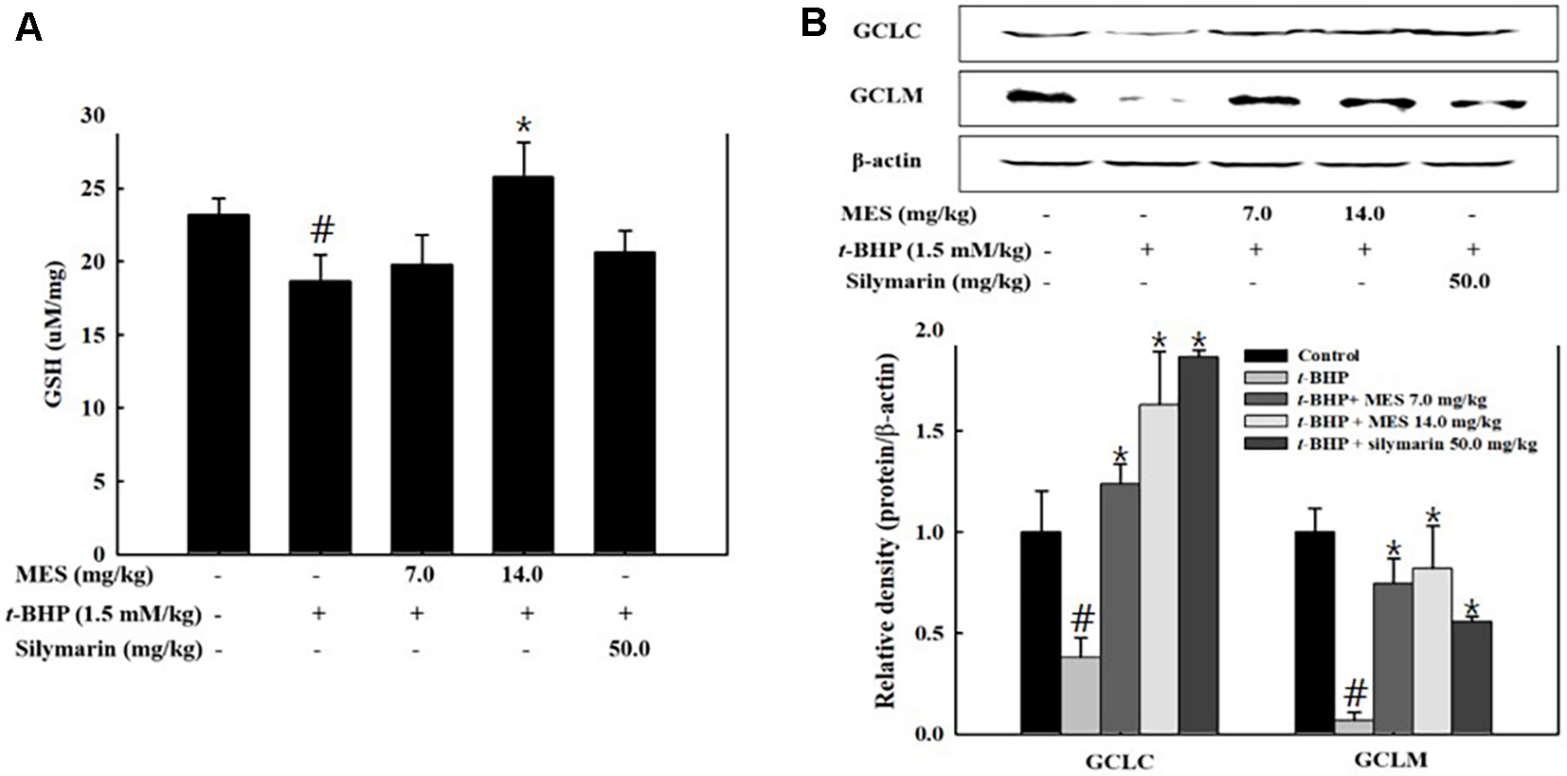
Effect of MES on GSH levels and GCLC, GCLM expression in liver tissue Mice (n = 6/group) were pretreated with MES or silymarin for 5 days before t-BHP (1.5 mM/kg, ip) treatment and subsequent liver damage induction. Post-sacrifice, GSH levels were measured with a glutathione assay kit (**A**) and expression levels of GCLC and GCLM were analyzed by Western blot (**B**). Data are means ± SD from seven mice. #P < 0.05 signifies significant difference from the control group not treated with t-BHP; *P < 0.05 signifies significant differences from the t-BHP only group.

GSH is a critical intracellular antioxidant essential for the antioxidative system under oxidative stress. To evaluate the impact of MES on GSH levels, liver tissue GSH concentrations were measured using an ELISA. Treatment with t-BHP significantly depleted GSH levels in the liver (Fig. 3A). Supplementation with 14.0 mg/kg of MES restored these levels to those of the control group. In contrast, the silymarin-treated group showed a slight recovery in GSH levels, though these were not statistically different from the t-BHP group (Fig. 3A). Glutamate-cysteine ligase (GCL), the rate-limiting enzyme in GSH synthesis, catalyzes the condensation of cysteine and glutamate to form gamma-glutamylcysteine. GCL consists of two subunits: glutamate-cysteine ligase catalytic (GCLC) and glutamate-cysteine ligase modifier (GCLM). Both subunits are crucial for the production of intracellular GSH. The expression levels of GCLC and GCLM were assessed by Western blot analysis. The results showed that t-BHP treatment suppressed the production of these enzymes, but their expression was restored by treatment with either MES or silymarin (Fig. 3B). This recovery suggests that the increase in GSH levels observed with MES and silymarin treatments is linked to enhanced GCL production.

### Effect of MES on Antioxidant Enzyme Activities

**Fig. 4:**
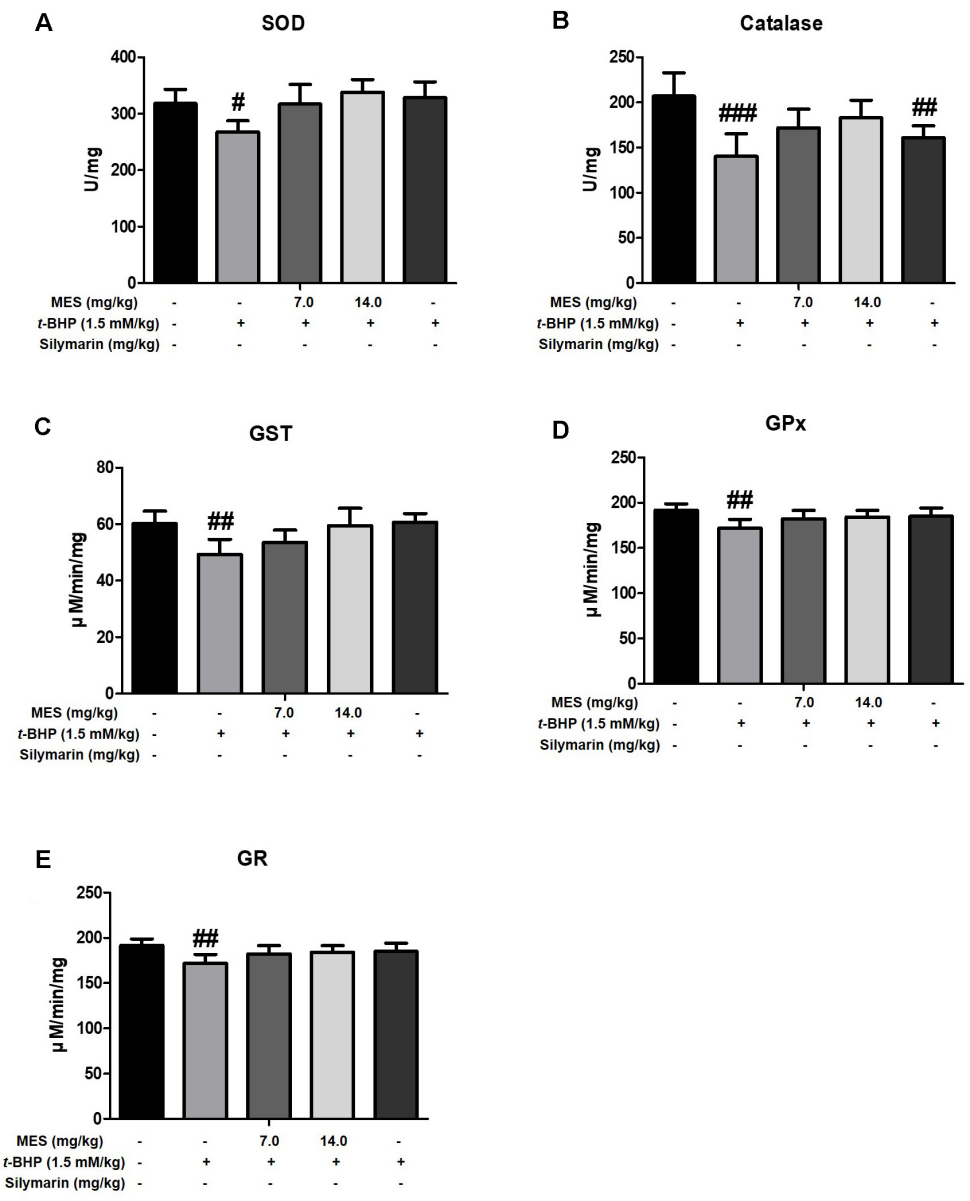
Impact of MES on antioxidant enzyme activities in t-BHP-induced liver damage Following 5 days of pretreatment with MES or silymarin, mice (n = 6/group) received t-BHP (1.5 mM/kg, ip). Antioxidative enzyme activities, including SOD (**A**), catalase (**B**), GST (**C**), GPx (**D**), and GR (**E**) in liver lysates were evaluated. Data represent means ± SD. #P < 0.05 and ##P < 0.01 indicate significant differences from the control group not treated with t-BHP. Abbreviations: SOD: superoxide dismutase, GST: glutathione S-transferase, GPx: glutathione peroxidase, GR: glutathione reductase.

**Fig. 5:**
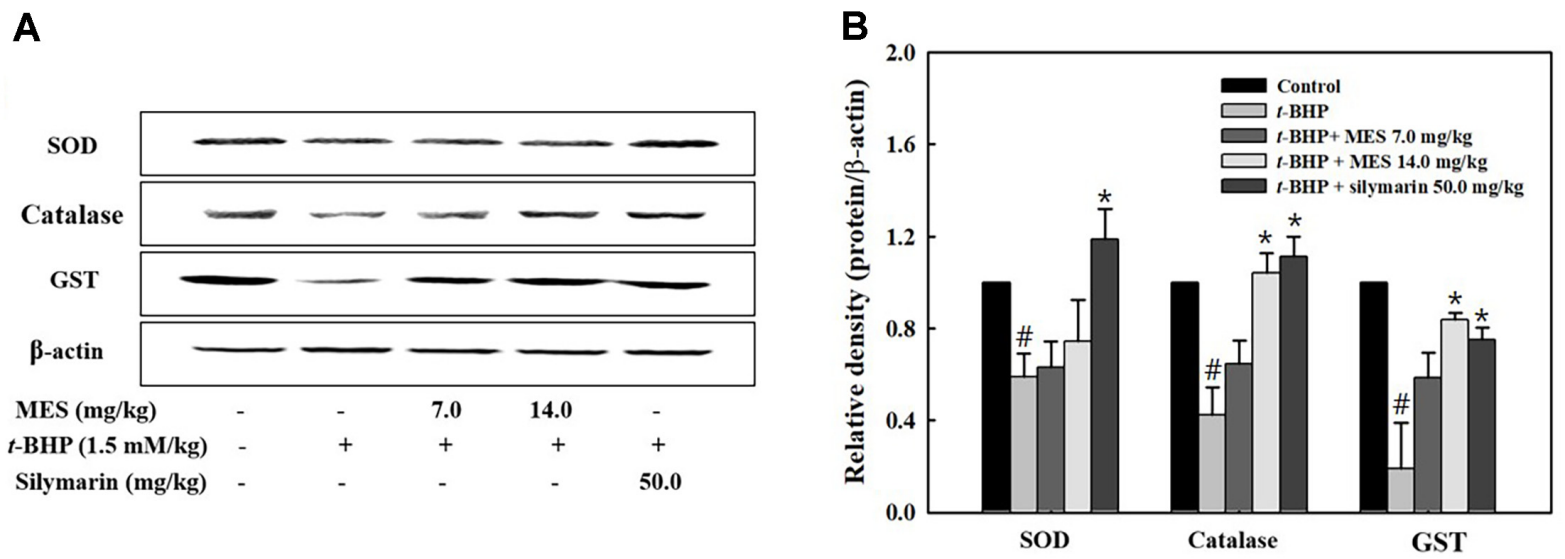
Effect of MES on antioxidant enzyme expression in liver tissue of mice (**A**) Expression levels of antioxidant enzymes were determined in liver tissue lysates by Western blot. (**B**) Quantification of Western blot bands using Image J software. Data represent means ± SD from seven mice. #P < 0.05 indicates a significant difference from the control group; *P < 0.05 indicates significant differences from the t-BHP only group.

Antioxidative enzymes such as SOD and catalase play critical roles in scavenging free radicals under oxidative stress. To assess the effect of MES on the activities of these antioxidative enzymes, SOD and catalase activities were measured in liver lysates. The t-BHP treatment group exhibited significantly decreased activities of both SOD and catalase compared to the control group (Fig. 4A and 4B). In contrast, the MES treatment group displayed enzyme activities comparable to those of the control group, indicating effective recovery (Fig. 4A and 4B). The silymarin treatment group showed a notable increase in SOD activity, although the increase in catalase activity was only slight (Fig. 4A and 4B). Protein expression of SOD and catalase paralleled the trends observed in enzyme activities. The production of both enzymes, which was suppressed in the t-BHP treatment group, was restored following treatment with either MES or silymarin (Fig. 5A and 5B).

### Effect of MES on GSH-Related Enzyme Activities

To evaluate the effect of MES on the activities of GSH-regenerating enzymes, activities of glutathione S-transferase (GST), GPx, and GR were measured in liver lysates. GST, which catalyzes the conjugation of the reduced form of GSH to xenobiotic substrates for detoxification, showed decreased activity in the t-BHP treatment group (Fig. 4C-4E). Conversely, both the MES and silymarin treatment groups maintained GST activity levels; notably, the 14.0 mg/kg MES group and the silymarin group displayed GST activity levels comparable to those of the control group (Fig. 4C-4E). The expression level of GST was decreased in the t-BHP group but was dose-dependently increased in the MES and silymarin groups (Fig. 5A and 5B).

GPx functions to reduce lipid hydroperoxides to their corresponding alcohols and to reduce free hydrogen peroxide to water. GR catalyzes the reduction of oxidized glutathione back to GSH, playing a crucial role in scavenging ROS and maintaining the cell's reduced environment. Both enzymes are vital for maintaining the redox homeostasis within cells. In the t-BHP treatment group, activities of both GPx and GR were decreased; however, these activities were partially restored in the MES and silymarin treatment groups, indicating effective recovery of these critical antioxidant defenses (Fig. 4C-4E).

### Effect of MES on the Activation of Nrf2

**Fig. 6:**
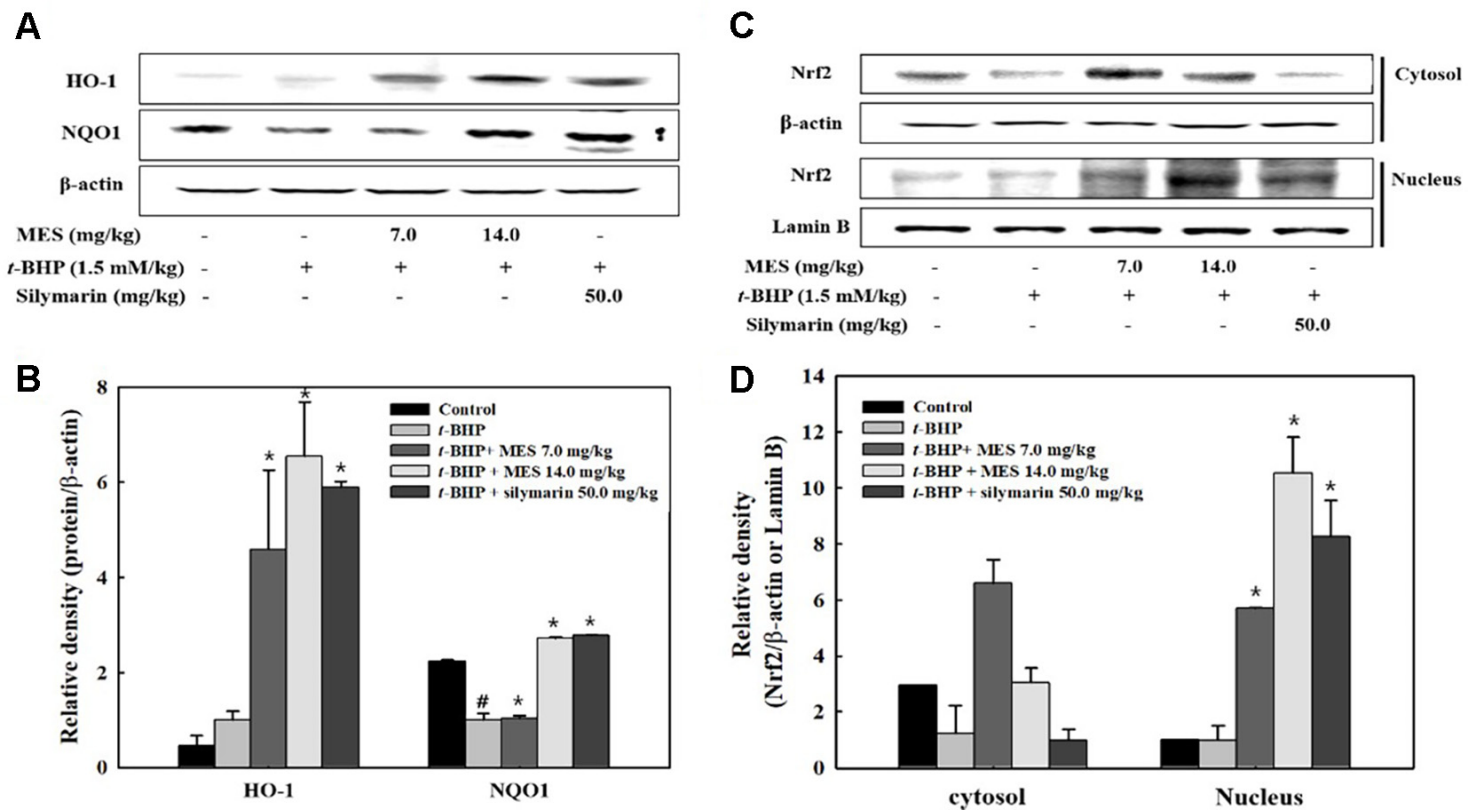
MES-mediated modulation of HO-1, NQO1, and Nrf2 expression in mouse liver tissue (**A**) HO-1 and NQO1 expressions were evaluated by Western blot in liver tissue lysates. (**B**) Nrf2 levels in cytosolic and nuclear fractions were also determined by Western blot. Values are shown as means ± SD from seven mice. #P < 0.05 indicates a significant difference from the control group; *P < 0.05 indicates significant differences from the t-BHP only group

Since Nrf2 is a critical transcription factor for regulating antioxidant and detoxification enzymes, the potential upregulating effects of MES on Nrf2 activation were investigated. Both MES and silymarin treatment groups exhibited elevated levels of Nrf2 in the nucleus compared with the control and t-BHP treatment groups, suggesting that MES and silymarin induce Nrf2 activation (Fig. 6C and 6D). In the cytosolic fraction, Nrf2 levels in the MES groups were higher than those in the t-BHP and control groups, with the 14.0 mg/kg MES group showing lower Nrf2 production than the 7.0 mg/kg MES group. The silymarin group displayed reduced cytosolic Nrf2 production compared to the t-BHP group (Fig. 6C and 6D). These results suggest that MES not only promotes Nrf2 production but also its activation, while silymarin mainly enhances Nrf2 activation without increasing its production.

Heme oxygenase-1 (HO-1) and NAD(P)H:quinone oxidoreductase 1 (NQO1) are detoxifying enzymes directly regulated by Nrf2. The expressions of HO-1 and NQO1 increased in a dose-dependent manner with MES treatment (Fig. 6A and 6B). The silymarin group exhibited similar expression levels of HO-1 and NQO1 as the 14.0 mg/kg MES group (Fig. 6A and 6B). These findings indicate that the expressions of HO-1 and NQO1 are associated with the activation of Nrf2.

## Discussion

This study provides evidence for the hepatoprotective effects of a MES derived from *S. serratifolium* in a model of t-BHP-induced liver injury in mice. t-BHP is extensively utilized as a pro-oxidant to induce acute hepatic oxidative stress, offering a model to explore protective strategies against oxidative damage [18, 19]. t-BHP is metabolized by cytochrome P450 enzymes in hepatocytes to generate toxic radicals, such as peroxyl and alkoxy radicals, leading to lipid peroxidation and alkylation of cellular macromolecules [20]. Another pathway involves GSH in its detoxification reaction, producing tert-butyl alcohol and oxidizing GSH, which can deplete cellular glutathione levels [21].

The protective properties of MES were demonstrated across multiple dimensions, including histopathological improvement, enzymatic regulation, enhancement of the antioxidant defense system, and modulation of the Nrf2 signaling pathway. Histologically, t-BHP induced severe hepatocellular damage, characterized by swelling, vacuolation of the cytoplasm, and necrosis, particularly in the portal regions. These manifestations are typical indicators of oxidative stress and inflammation [22]. The portal region is particularly vulnerable due to its role as the entry point for blood from the portal vein, which carries absorbed nutrients, toxins, and xenobiotics from the gastrointestinal tract. This makes hepatocytes in this region more exposed to oxidative insults and metabolic stress [23]. Remarkably, MES treatment significantly ameliorated these pathological changes, reducing hepatocyte swelling and cytoplasmic vacuolation, suggesting that MES components possess potent anti-inflammatory and cytoprotective properties that help preserve cellular integrity against chemically induced liver damage.

Biochemically, MES significantly reduced elevated levels of serum hepatic enzymes (ALT, AST, and LDH), which are indicative of hepatic injury and reflect damage to the liver architecture. This effect was particularly notable at higher dosages of MES, suggesting a dose-dependent response in mitigating hepatic damage.

Moreover, MES played a crucial role in enhancing the liver's antioxidant capacity. It effectively countered oxidative stress, evidenced by decreased lipid peroxidation (MDA levels). The treatment improved the activities of key antioxidant enzymes such as SOD and catalase, crucial for detoxifying ROS. Additionally, MES restored GSH levels that were depleted by t-BHP treatment, enhancing the primary defense system against oxidative stress. The activities of GSH-regenerating enzymes like GPx and GR were also restored, thereby stabilizing the cellular redox state [24].

One of the most significant findings was the activation of the Nrf2 pathway by MES. Nrf2 is a major transcription factor that regulates the expression of various detoxifying and antioxidative genes [25]. Under basal conditions, Nrf2 is sequestered in the cytoplasm by its repressor, Kelch-like ECH-associated protein 1 (Keap1), which facilitates its ubiquitination and proteasomal degradation. However, upon exposure to oxidative stress, conformational changes in Keap1 prevent Nrf2 degradation, allowing Nrf2 to accumulate and translocate into the nucleus. There, it binds to antioxidant response elements in the promoter regions of target genes, initiating the transcription of phase II detoxifying and antioxidant enzymes [26, 27]. Elevated levels of Nrf2 in the nucleus following MES treatment indicate enhanced transcriptional activity, leading to increased production of protective enzymes such as HO-1 and NAD(P)H:NQO1. These enzymes are critical for the hepatic response to oxidative stress and chemical insults, suggesting that the activation of Nrf2 by MES not only contributes to direct detoxification of harmful agents but also enhances the cellular antioxidant response.

Previous studies have shown that MES exhibits strong antioxidant activities, with major components identified as SHQA, SCM, and SQA. The hepatoprotective effect of MES on t-BHP-induced HepG2 cells was attributed to both direct scavenging of ROS and the induction of antioxidant enzyme activities [28, 29]. Among these, SHQA and SCM have been particularly noted for their bioactivity. SHQA has demonstrated potent anti-inflammatory properties by inhibiting nitric oxide production and suppressing the expression of inflammatory mediators in activated macrophages. Likewise, SCM has been shown to exert hepatoprotective effects by activating antioxidant enzymes and attenuating oxidative stress in cellular models. These properties suggest that the observed *in vivo* efficacy of MES may, in part, be attributed to the synergistic activity of these key meroterpenoids [30].

While silymarin served as an effective positive control with known hepatoprotective properties, MES displayed comparable or even superior effects in certain assays. This suggests that the meroterpenoids in MES might offer unique or more potent protective mechanisms, warranting further investigation to fully harness their therapeutic potential and elucidate their molecular mechanisms.

Overall, the results of this study demonstrate that the meroterpenoid-rich fraction from *S. serratifolium* provides robust protection against t-BHP-induced hepatic injury. These protective effects stem from reduced oxidative damage, enhanced antioxidant defense, and Nrf2 activation. The findings underscore the therapeutic potential of marine-derived natural products in treating liver diseases and suggest that MES could be a promising candidate for further development as a hepatoprotective agent. Future studies should aim to isolate specific active compounds within MES and elucidate their mechanisms of action to fully harness their therapeutic potential.
